# Current knowledge about pyruvate supplementation: A brief review

**DOI:** 10.1016/j.smhs.2024.02.007

**Published:** 2024-02-28

**Authors:** Robert A. Olek, Sylwester Kujach, Zsolt Radak

**Affiliations:** a Department of Athletics, Strength, and Conditioning, Poznan University of Physical Education, Poznan, Poland; b Department of Neurophysiology, Neuropsychology and Neuroinformatics, Medical University of Gdansk, Gdansk, Poland; c Research Institute of Sport Science, Hungarian University of Sport Science, Budapest, Hungary; d Faculty of Sport Sciences, Waseda University, Tokorozawa, Japan

**Keywords:** Acidosis, Bicarbonate, Lactate, Nicotinamide adenine dinucleotide, Sirtuins

## Abstract

Pyruvate is a three-carbon ketoacid that occurs naturally in cells. It is produced through enzymatic reactions in the glycolytic pathway and plays a crucial role in energy metabolism. Despite promising early results, later well-controlled studies of physically active people have shown that pyruvate supplementation lasting more than 1 week has no ergogenic effects. However, some data suggest that ingested pyruvate may be preferentially metabolized without accumulation in the bloodstream. Pyruvate exhibits antioxidant activity and can affect the cellular redox state, and exogenous pyruvate can influence metabolism by affecting the acid-base balance of the blood. This brief review focuses on the potential effects of pyruvate as a supplement for active people. The current state of understanding suggests that studies of the effects of pyruvate supplementation should prioritize investigating the timing of pyruvate intake.

## Abbreviation list

AD: Alzheimer disease
ALA: alanine
BDNF: brain-derived neurotrophic factor
CaPYR: calcium pyruvate
d: days
EtPYR: ethyl pyruvate
GPT: glutamate-pyruvate transaminase
GSH: glutathione
GSSG: glutathione disulfide
ip: intraperitoneally
iv: intravenously
LA: lactate
LDH: lactate dehydrogenase
ME: malic enzyme
NAD^+^: nicotinamide adenine dinucleotide
NADH: nicotinamide adenine dinucleotide reduced form
NADPH: nicotinamide adenine dinucleotide phosphate reduced form
NaPYR: sodium pyruvate
OAA: oxaloacetate
PYR: pyruvate
sc: subcutaneously
SIRTs: sirtuins - NAD-dependent mitochondrial deacetylases family
TCA: tricarboxylic acid
$\dot{V}O_{2}$max: maximal oxygen consumption
wk: weeks

## 1 Introduction

Pyruvate (PYR) plays an important role in the major mammalian metabolic pathways. PYR occupies a central position between the catabolic and anabolic pathways involved in the metabolism of carbohydrates, fats, and amino acids (for details see^1^^,^^2^). A closer examination of these pathways indicates that, in most cases, PYR enters the mitochondria before further metabolism. Within mitochondria, PYR may be converted by the pyruvate dehydrogenase complex to acetyl-CoA, which can enter the tricarboxylic acid (TCA) cycle, or it may serve as the starting point for the synthesis of long chain fatty acids, steroids, and ketone bodies. PYR may be used to produce alanine (ALA) via the action of glutamate-pyruvate transaminase (GPT), and may thus play an important role in maintaining the levels of the TCA cycle intermediate, α-ketoglutarate. In the cytoplasm, PYR can be reduced to lactate (LA) via LA dehydrogenase (LDH) or to malate via decarboxylating malate dehydrogenase (malic enzyme: ME). In both cases, the reduced form of nicotinamide adenine dinucleotide (NADH) is oxidized to NAD^+^. Therefore, PYR may affect the NADH/NAD^+^ ratio.

## 2 Effects of prolonged PYR supplementation on body weight and composition

**Fig. 1:**
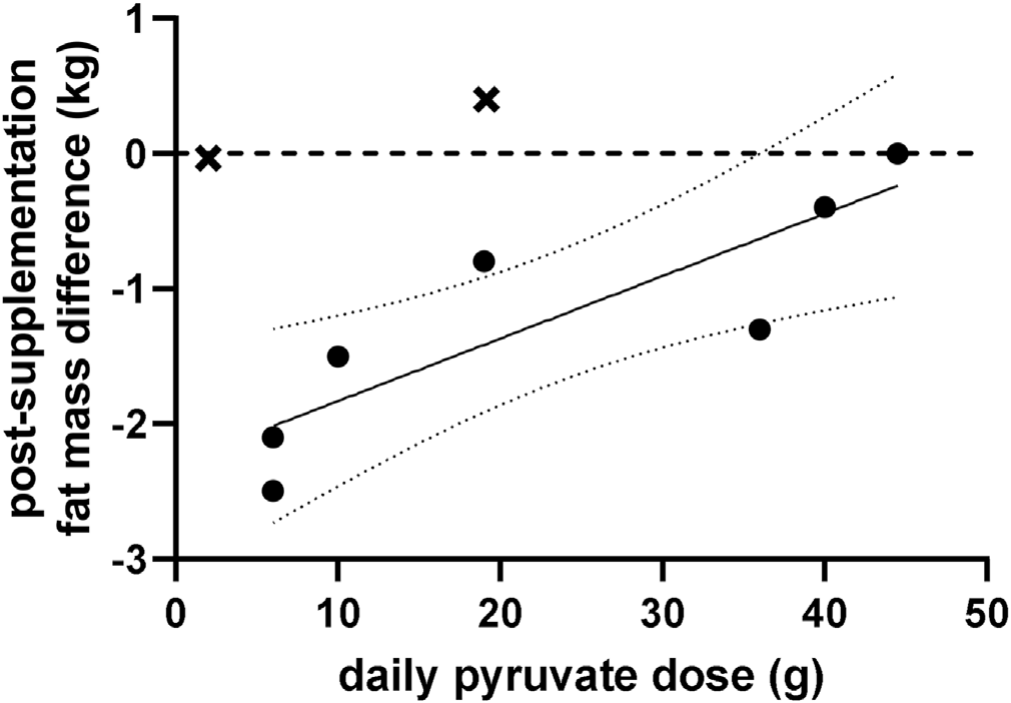
Daily dose of PYR supplementation effect on body fat loss. ( ​× ​) studies on athletes with low body fat content excluded from the analysis; dotted lines represent 95% confidence interval.

The research findings from studies of PYR supplementation as a weight loss aid are summarized in Table 1. The first studies by Stanko et al.,^3^, ^4^, ^5^, ^6^ showed that prolonged PYR supplementation decreased body weight by significantly reducing body fat mass. These studies, used high doses of PYR (16–53 ​g/d) along with dihydroxyacetone and/or energy restriction.^3^, ^4^, ^5^, ^6^ Subsequent studies have examined lower doses of PYR supplementation (2–10 ​g/d) and found conflicting results.^7^^,^^8^ The meta-analysis of six randomized clinical trials showed that PYR was not consistently effective in reducing body weight.^9^ Two of the studies that found PYR supplementation to be ineffective involved trained athletes who had low body fat composition at the start of the study (15.7% for American football players^10^ and 8.8% for soccer players^11^), and fat loss is unlikely to occur in such lean active people. After excluding the studies involving athletes, we observed inverse relationship between the changes in fat loss and the daily PYR dose (Fig. 1). Because PYR may accelerate fatty acid synthesis through acetyl-CoA, excessive PYR intake may be ineffective in promoting fat loss.

**Table 1:** Main results of PYR supplementation on weight loss studies.

participants female/male age	intervention	period (days)	pyruvate form	average pyruvate dose (g/d)	placebo	body mass change in pyruvate group (kg)	body mass change in *placebo* group (kg)	difference between changes in body mass	Ref. #
obese 14/0 not reported	confined to bed 1 015 ​kcal/day	21	20 ​g NaPYR +16 ​g CaPYR	36	iso-energetically polyglucose	−5.9 ​± ​0.7	−4.3 ​± ​0.3	−1.6 ​kg *p* ​< ​0.05	3
obese 13/0 PYR 48.4 ​± ​3.2 PLA 48.7 ​± ​4.9	confined to bed 501 ​kcal/day	21	10 ​g NaPYR +9 ​g CaPYR *+* 12 ​g DHAP	19	iso-energetically polyglucose	−6.5 ​± ​0.3	−5.6 ​± ​0.2	−0.9 ​kg *p* ​< ​0.05	4
hyperlipidemic patients 31/9 PYR 56.3 ​± ​2.5 PLA 54.8 ​± ​2.8	high-fat anabolic (26.3–28.7 ​kcal/kg/day) diet	42	18.8–30 ​g NaPYR +16.8–23 ​g CaPYR	44.5	iso-energetically polyglucose	0.6 ​± ​0.2	0.7 ​± ​0.2	−0.1 ​kg N.S.	5
hyperlipidemic patients 25/9 PYR 58 ​± ​3 PLA 56 ​± ​3	low-cholesterol (21.5 ​kcal/kg/day) diet	42	14–28 ​g NaPYR +13–25 ​g CaPYR	40	iso-energetically polyglucose	−0.7 ​± ​0.2	−0.1 ​± ​0.2	−0.6 ​kg *p* ​< ​0.05	6
healthy BMI >25 16/20 PYR 37.1 ​± ​3.2 PLA 35.6 ​± ​2.5	2 000 ​kcal/day +45min exercise (3/wk)	42	6 ​g NaPYR + CaPYR	6	maltodextrin	not reported	not reported	N.S.	12
healthy BMI >25 16/10 PYR 36.5 ​± ​3.0 PLA 39.9 ​± ​3.8	2 000 ​kcal/day + 45min exercise (3/wk)	42	6 ​g	6	maltodextrin	−1.2	0.0	−1.2 ​kg N.D.	7
moderately overweight untrained 23/0 33 ​± ​8	resistance training 3/wk, walking 30min. 3/wk	30	10 ​g CaPYR	10	CaCO_3_ maltodextrindextrose	0.3	1.2	−0.9 ​kg *p* ​< ​0.05	8
American football players 0/22 PYR 18.6 ​± ​0.6 PLA 18.3 ​± ​0.4	weight training (3/wk) football practice (2–3/wk)	35	0.22 ​g/kg CaPYR	19	silica	0.0	0.0	0.0 ​kg N.S.	10
soccer players 0/22 PYR 22.5 ​± ​2.3 PLA 21.9 ​± ​3.1	specific training program	28	2 ​g	2	cellulose	−1.1	−0.7	−0.4 ​kg N.S.	11

PYR – pyruvate; PLA – placebo; NaPYR – sodium pyruvate; CaPYR – calcium pyruvate; DHAP – dihydroxyacetone phosphate; N.S. – not significant; N.D. – not determined.

## 3 Supplementation and exercise performance

The first studies of PYR supplementation and exercise performance were also conducted by Stanko et al.^13^^,^^14^ These authors found improvements in aerobic endurance capacity after PYR supplementation, possibly as a result of an increased rate of muscle glucose uptake and sparing of muscle glycogen. However, these studies involved untrained participants who consumed 25 ​g of PYR per day combined with 75 ​g dihydroxyacetone. Later studies reported different results.^10^^,^^15^^,^^16^ In one study, 5 weeks of PYR supplementation (0.22 ​g/kg/d) during the training program of American football players did not improve maximal strength, cycle ergometer peak power, and static vertical jump power output.^10^ Similarly, 2 weeks of PYR supplementation (8.1 ​g/d) did not improve the ability to maintain power output without fatigue, which is defined as critical power.^15^ In another study, 1 week of PYR supplementation (7 ​g/d) did not influence the time to exhaustion during exercise performed at 75%–80% $\dot{V}$O_2_max in highly trained cyclists ($\dot{V}$O_2_max [62.3 ​± ​3.3] ​ml O_2_/kg/min.).^16^ These findings have led to a loss of interest in PYR supplementation by athletes.^17^

A recent study reported higher blood pH, bicarbonate level, and base excess, as well as improved performance during high-intensity interval exercise after PYR supplementation. The study involved male soccer players aged (20 ​± ​2) years (body fat 13.1% ​± ​3.5%) with at least 5 years of training experience and $\dot{V}$O_2_max (55.9 ​± ​5.4) ​ml/kg/min of O_2_.^18^ The supplementation protocol lasted for 1 week and the dose was 0.1 ​g/kg/d, which provides about 7 ​g/d of PYR.^18^ One major difference in the supplementation protocol may explain the differences in results between this study and those mentioned above: on day 7 of the more recent study, the entire daily dose was ingested at least 60 ​min before the exercise test started.^18^ It is possible that the timing of PYR intake is a crucial variable, this point was not considered in the previous studies.^10^^,^^13^, ^14^, ^15^

## 4 Single-dose PYR ingestion

Morrison et al.^16^ measured whole-blood and plasma PYR levels for the 4-h period following various single-dose PYR consumption and found no effect of 7, 15, and 25 ​g of PYR. The inability to detect any elevation of PYR level in the blood, as well as increased borborygmus and ﬂatulence in subjects consuming higher doses of PYR, led the authors to the speculation that PYR may be decarboxylated in the gastrointestinal tract or eliminated through the feces, and not delivered into muscle cells.^16^

Supplemental PYR may be absorbed by the intestinal epithelium and transported via the portal vein to the liver, where hepatocytes may utilize PYR for gluconeogenesis,^19^ which would not affect circulating PYR levels. Blood glucose level is also not significantly affected by various PYR doses.^16^ However, in another study, an increase in the resting respiratory exchange ratio 3 ​h after a single oral intake of PYR suggested greater carbohydrate oxidation.^20^ In addition, the increase in plasma free fatty acid level observed in placebo was attenuated 3 ​h after acute ingestion of 7 ​g of PYR^20^ and 4 ​h after intake of 25 ​g of PYR compared with the 7 ​g dose.^16^ These findings suggest that PYR may be used as a preferential energy source in the human body without increasing blood PYR levels.

**Fig. 2:**
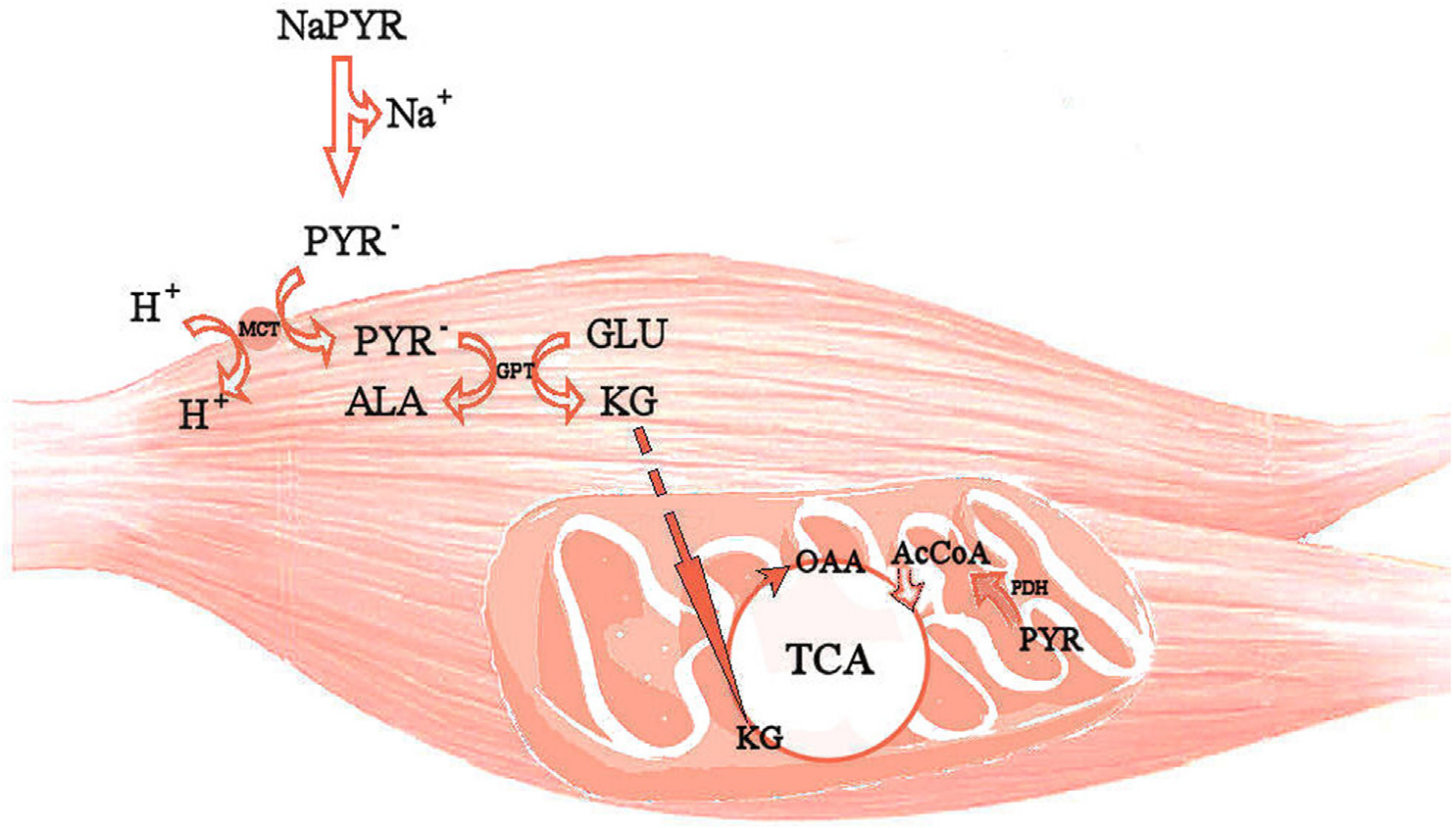
Hypothetical metabolism of exogenous PYR at rest; PYR – pyruvate; ALA – alanine; GLU – glutamate; KG –-ketoglutarate; GPT – glutamate-pyruvate transaminase; OAA – oxaloacetate; AcCoA – acetylCoA; PDH – pyruvate dehydrogenase; TCA – tricarboxylic acid cycle; MCT – monocarboxylate transporter.

The rate-limiting step of citrate formation in the TCA cycle is the low concentration of oxaloacetate (OAA) in mitochondria,^21^ and it is possible that PYR could be used to replenish mitochondrial OAA levels. In nonmuscle cells, PYR can be carboxylated to OAA, and may play a role in the muscles in supporting OAA formation by the generation of greater α-ketoglutarate concentration via GPT (Fig. 2).^1^ Intravenous PYR infusion increases the levels of TCA cycle intermediates, mainly malate, in skeletal muscles.^22^

The uptake of PYR by cells depends on the monocarboxylate transporter system.^23^ This system is located in the plasma membrane and transports monocarboxylates together with H^+^ (Fig. 2), which may indirectly spare blood bicarbonate and increase blood pH.^24^ Because of sodium-coupled transport, the effects of sodium PYR (NaPYR) differ from those of calcium PYR (CaPYR) in terms of their alkalosis-inducing effects.^20^ The buffering property of NaPYR has been described in an intravenous infusion study,^25^ although intravenous PYR infusion has been found to be ineffective in increasing muscle PYR content.^22^ Moreover, single oral PYR intake causes an increase in blood pH, bicarbonate level, and base excess.^20^^,^^26^

A recently reported PYR supplementation protocol did not modify aerobic energy contributions during high-intensity interval exercises,^18^ a finding that agrees with previously published results.^16^ In the recent study, no changes were observed in the contributions of glycolytic energy after PYR supplementation despite modification of the blood buffering capacity. Interestingly, PYR supplementation improved phosphagen energy system regeneration during four sessions of 1-min cycling at 110% Wmax, interspersed with 1 min recovery periods, and six sessions of 6 s maximal cycling sprints, interspersed with 24 s passive recovery periods.^18^ In addition, peak power output and mean power output increased. Although, supraphysiological PYR concentration in a perfusing solution increases phosphagen levels and improves contractile properties of stressed myocardium,^27^ there is no evidence that oral PYR supplementation, even in the form of creatine-PYR, affects muscle creatine content and/or performance^10^^,^^15^ (this topic has been reviewed in detail elsewhere^28^^,^^29^). Therefore, it has been suggested that improved exercise performance may be achieved by increased phosphocreatine resynthesis and muscle contraction through a decrease in H^+^ concentration.^18^ However, PYR supplementation at a higher dose, and for a longer period does not improve explosive power,^10^ or delay fatigue.^15^ The resulting differences between studies may be explained by alkalization induced by a PYR dose ingested 60 ​min before the exercise tests.^18^ Alternatively, different forms of PYR may also explain resulting differences between studies. For example, NaPYR supplementation at a dose of 0.10 ​g/kg/d for 1 week is ergogenic,^18^ whereas CaPYR at a dose of 0.22 ​g/kg/d for 5 weeks is not,^10^ possibly because of the different alkalosis-inducing effects.^20^

## 5 Redox state modulation

The nonenzymatic reaction of PYR with hydrogen peroxide was described more than 100 years ago,^30^ as follows:

CH_3_COCOO^−^ ​+ ​H_2_O_2_ → CH_3_COO^−^ ​+ ​CO_2_ ​+ ​H_2_O

Later studies confirmed the PYR antioxidant properties in various models^31^, ^32^, ^33^, ^34^ and indicated the direct scavenging potential toward peroxynitrite,^35^ hydroxyl radical,^36^ and superoxide anion radical.^37^ In addition to the antioxidant properties of PYR, other mechanisms of PYR stress-ameliorating effects have been proposed. These include an increase in sarcoplasmic reticular Ca^2+^ transport, improvement in mitochondrial function, increase in ATP concentration, or support of NADPH production to maintain the glutathione/glutathione disulfide (GSH/GSSG) redox potential (for review, see^27^^,^^38^). Another possible mechanism of PYR-induced protection is the modulation of cellular redox potential by decreasing the cytosolic NADH/NAD ​^+^ ​ratio.^39^

**Fig. 3:**
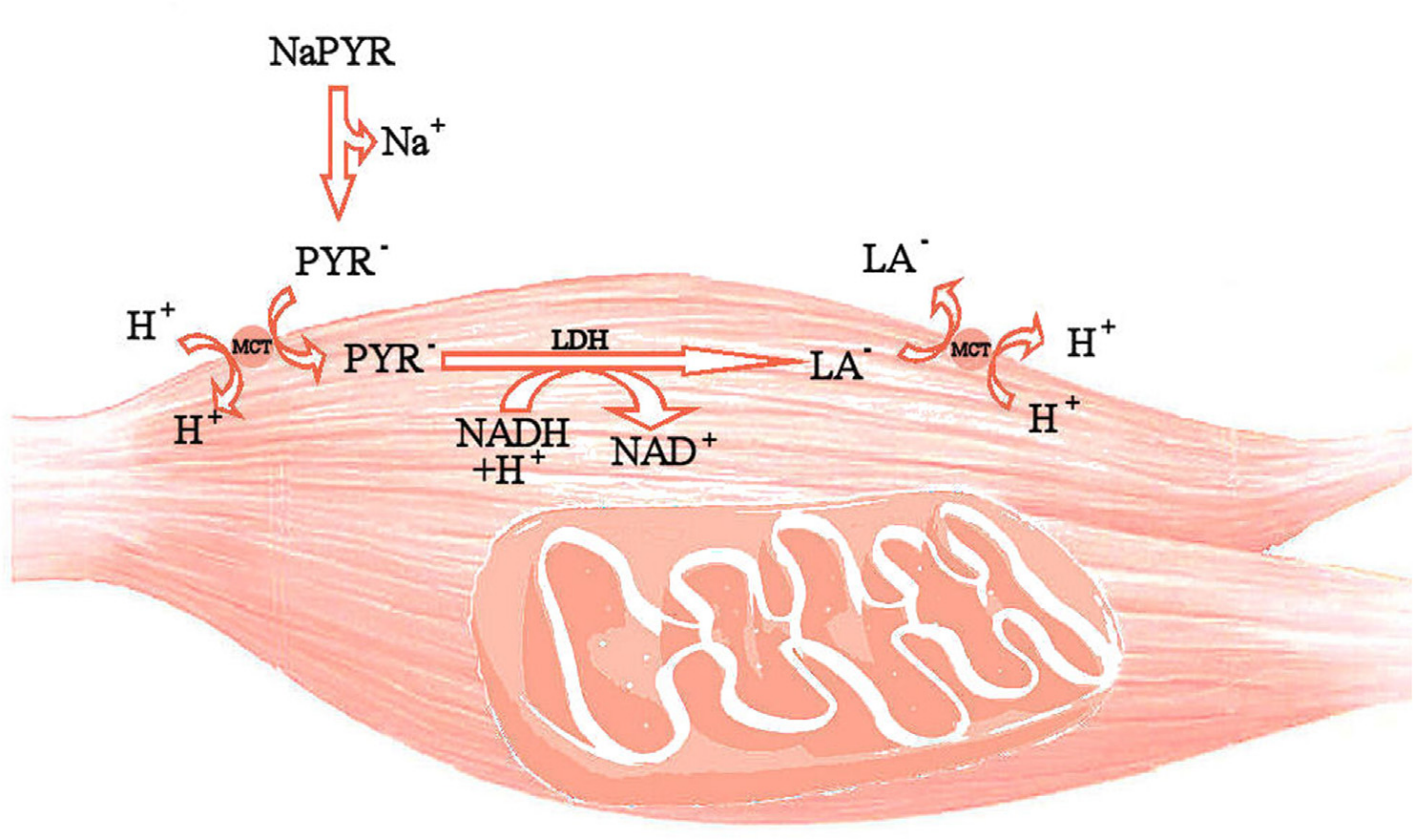
Hypothetical metabolism of exogenous PYR during exercise at 90% $\dot{V}$O_2_max. PYR – pyruvate; LA – lactate; LDH – lactate dehydrogenase; MCT – monocarboxylate transporter.

In our study, a single-dose PYR ingestion caused a greater increase in blood LA concentration after exercise at 90% $\dot{V}$O_2_max compared with placebo.^26^ This result may reflect elevated blood bicarbonate concentration.^20^^,^^26^ It is possible that a higher LA concentration may also be caused by the reaction of PYR with accumulated NADH. In muscle cells, NADH is oxidized continuously by the malate-aspartate shuttle or by LDH.^40^ During exercise at a higher intensity, the rate of anaerobic glycolysis and the concentration of NADH in cytosol increase.^41^ Therefore, ingestion of PYR may not modify blood LA concentration at rest^16^ but may affect the redox state of muscle cells and accelerate LA production during exercise at higher power output (Fig. 3).^26^

## 6 Mechanisms of adaptation

The recognition of NAD^+^ as a multifunctional signaling molecule has been driven mainly by research in the field of exercise and nutrition. One of the key cell signaling candidates proposed is the NAD-dependent mitochondrial deacetylases family – sirtuins (SIRTs). SIRTs modulate many cellular processes, including energy metabolism, mitochondrial biogenesis, and protection against oxidative stress (these topics have been reviewed in detail elsewhere^42^, ^43^, ^44^).

Because NAD^+^ is a coenzyme of the reaction catalyzed by SIRTs, the activity of these enzymes increases when NAD^+^ concentration increases. By contrast, the expression follows a different pattern; that is, the enzyme expression is induced by an increase in the NADH/NAD^+^ ratio (predominantly a simultaneous decrease in NAD^+^ and increase in NADH concentrations).^45^ Physical exercise modulates the redox state of muscle cells according to the exercise intensity. During low or moderate exercise intensity, cytosolic NADH is continuously oxidized by the malate-aspartate shuttle, which controls the NADH/NAD ​^+^ ​ratio.^40^ At higher exercise intensity, the accumulation of cytosolic NADH can constrain glycolysis and thus limit performance.^40^ Therefore, a higher turnover via LDH is necessary to restore the NADH/NAD^+^ ratio. The modulation of the NADH/NAD^+^ ratio by exogenous PYR may impact histone deacetylase activity but only during exercise performed at intensities higher than the LA threshold.

## 7 PYR and neuroprotection

The brain is a highly energy-demanding organ, and its proper functioning depends on an adequate supply of energy substrates.^46^^,^^47^ As a source of energy, PYR has a positive effect in animal models of Alzheimer disease (AD).^48^, ^49^, ^50^ A diet enriched with PYR and β-hydroxybutyrate for 5 weeks, averaging ∼26 ​mg daily intake of substrates, improved cerebral energy metabolism in transgenic mice. This involved mitigating glycogen depletion and NAD(P)H autofluorescence.^48^ Subsequent study on PYR alone confirmed increased brain glycogen storages, and indicated elevation of energy metabolites, such as creatine, LA, and glutamate.^49^ Koivisto and colleagues^49^ demonstrated that long-term NaPYR supplementation (∼800 ​mg/kg/d for 2–6 months) increased exploratory behavior in both wild-type and AD transgenic mice, highlighting its effects on cognitive function.

In addition to its well-recognized function in energy metabolism, PYR may be an effective neuroprotector to reduce the rate of cognitive decline.^51^ A recent study reported that the intraperitoneal injection of ethyl pyruvate (EtPYR) promoted the expression of brain-derived neurotrophic factor (BDNF) by astrocytes and astrocyte transdifferentiation into oligodendrocytes, which suggested that PYR treatment may help to facilitate myelin sheath regeneration.^52^ Given that triggering BDNF expression in the brain is recognized as having neuroprotective effects,^53^ BDNF stimulation by PYR may have neuroprotective properties and improve cognitive performance.

PYR acts as both an antioxidant and anti-inflammatory compound, and may protect neurons from oxidative stress and neuroinflammation, both of which are associated with cognitive decline and neurodegenerative disorders.^49^^,^^54^ Systemic administration of PYR has been reported to have neuroprotective effects in animal models of brain injury,^55^ hypoglycemic cognitive impairment,^56^ ethanol-induced neurodegeneration,^57^ and age-dependent cognitive deficits in a mouse model of AD.^50^ The results of *in vivo* studies showing a neuroprotective effect of PYR are summarized in Table 2. In addition, in vitro studies have shown that PYR has protective effects against glutamate neurotoxicity,^58^ neuronal cell death induced by hydrogen peroxide,^59^ oxygen-glucose deprivation,^60^ and zinc-induced cortical neuronal death.^61^

**Table 2:** Main results of PYR neuroprotective studies.

animals model	intervention	administration	average pyruvate dose	period	pyruvate form	outcome	Ref. #
closed head injury rats	acute	iv	0.9 mmoles/100 ​g	–	PYR	improved neurological outcome	62
traumatic brain injury rats	acute	iv	infusion over 30 ​min of 1 ml/100 ​g 1 ​M	–	PYR	hippocampal neuron survival	63
status epilepticus rats	acute	ip	250 ​mg/kg	–	PYR	hippocampal neuron lose prevented	64
cortical contusion injury in rats	acute	ip	500 ​mg/kg 1 000 ​mg/kg 3 ​× ​1 000 ​mg/kg	–	NaPYR	attenuate cortical cell damage	55
ischemic-middle cerebral artery occlusion rats	acute	ip	500 ​mg/kg	–	EtPYR	protective anti-inflammatory action	65
focal ischemia rats	acute	ip iv	62.5–250 ​mg/kg	–	NaPYR	neuroprotective capacity in focal cerebral ischemia	66
insulin-induced hypoglycemia rats	acute	ip	500 ​mg/kg	–	NaPYR	reduces the neuronal death and cognitive impairment	56
ethanol injected C57Bl/6 mice	acute	sc	500 ​mg/kg	–	PYR	reduced neuronal cell loss in the cortex and thalamus	57
C57Bl/6J male mice	acute	ip	500 ​mg/kg	–	NaPYR	no effect in the passive avoidance task	49
3 month old 3xTg-Alzheimer Disease mice model	chronic	ip	500 ​mg/kg	3× week per 9 months	PYR	counteract progression of AD-related cognitive deficits and neuronal loss	50
cuprizone-induced demyelination mice model	chronic	ip	10 ​mg/kg	14 days	EtPYR	improved behavioural performance and promoted myelin regeneration	67
cuprizone-induced demyelination mice model	chronic	ip	20 ​mg/kg	14 days	EtPYR	promoted astrocytes to phagocytized myelin debris for removing the harmful substances of myelin regeneration, BDNF and CNTF induction	52

iv – intravenously; ip – intraperitoneally; sc – subcutaneously; PYR – pyruvate; NaPYR – sodium pyruvate; EtPYR - ethyl pyruvate; BDNF – brain-derived neurotrophic factor.

## 8 Future directions

Further research is warranted to elucidate the optimal timing, dosage, and form of PYR supplementation for maximizing its potential benefits on exercise performance, body composition, and metabolic health.

Deeper mechanistic studies are needed to unravel the molecular pathways underlying PYR's diverse effects on metabolism, cellular redox state, and exercise physiology. This includes investigating its interactions with key enzymes, signaling molecules, and metabolic pathways involved in energy production, antioxidant defense, and cellular signaling.

Conducting well-designed studies in specific populations, such as individuals with obesity, metabolic disorders, neurodegenerative diseases, or athletes, could provide valuable insights into the efficacy of PYR supplementation in diverse contexts.

Given the emerging role of gut microbiota in modulating host metabolism and health, investigating the impact of PYR supplementation on gut microbiota composition and function could provide novel insights into its metabolic effects and potential mechanisms of action.

## 9 Conclusions

PYR is an important compound in aerobic and anaerobic energy metabolism. PYR ingested before high-intensity exercise can affect power output. Prolonged supplementation, especially in combination with interval training, may induce adaptive changes in skeletal muscle metabolism and/or affect cognitive function through neuroprotective effects. It may be of interest to study the effects of PYR supplementation by focusing on the optimal timing, dosage, and form of PYR intake.

## Submission statement

The manuscript has not been published previously, is not under consideration for publication elsewhere, and is approved by all authors. If accepted, it will not be published elsewhere, including electronically in the same form, in English or any other language, without the written consent of the copyright holder.

## Authors' contributions

**Robert A. Olek:** Writing – original draft, Conceptualization. **Sylwester Kujach:** Writing – original draft. **Zsolt Radak:** Writing – review & editing.

## Conflict of interest

Zsolt Radak is ​an editorial board member for Sports Medicine and Health Science and was not in the editorial review or the decision to publish this article. Otherwise authors declare that they have no competing financial interests or personal relationships that could have appeared to influence the work reported in this paper.

## References

[1] Mole P.A., Baldwin K.M., Terjung R.L., Holloszy J.O. (1973). Enzymatic pathways of pyruvate metabolism in skeletal muscle: adaptations to exercise. Am J Physiol.

[2] Gray L.R., Tompkins S.C., Taylor E.B. (2014). Regulation of pyruvate metabolism and human disease. Cell Mol Life Sci.

[3] Stanko R.T., Tietze D.L., Arch J.E. (1992). Body composition, energy utilization, and nitrogen metabolism with a 4.25-MJ/d low-energy diet supplemented with pyruvate. Am J Clin Nutr.

[4] Stanko R.T., Tietze D.L., Arch J.E. (1992). Body composition, energy utilization, and nitrogen metabolism with a severely restricted diet supplemented with dihydroxyacetone and pyruvate. Am J Clin Nutr.

[5] Stanko R.T., Reynolds H.R., Lonchar K.D., Arch J.E. (1992). Plasma lipid concentrations in hyperlipidemic patients consuming a high-fat diet supplemented with pyruvate for 6 wk. Am J Clin Nutr.

[6] Stanko R.T., Reynolds H.R., Hoyson R., Janosky J.E., Wolf R. (1994). Pyruvate supplementation of a low-cholesterol, low-fat diet: effects on plasma lipid concentrations and body composition in hyperlipidemic patients. Am J Clin Nutr.

[7] Kalman D., Colker C.M., Wilets I., Roufs J.B., Antonio J. (1999). The effects of pyruvate supplementation on body composition in overweight individuals. Nutrition.

[8] Koh-Banerjee P.K., Ferreira M.P., Greenwood M. (2005). Effects of calcium pyruvate supplementation during training on body composition, exercise capacity, and metabolic responses to exercise. Nutrition.

[9] Onakpoya I., Hunt K., Wider B., Ernst E. (2014). Pyruvate supplementation for weight loss: a systematic review and meta-analysis of randomized clinical trials. Crit Rev Food Sci Nutr.

[10] Stone M.H., Sanborn K., Smith L.L. (1999). Effects of in-season (5 weeks) creatine and pyruvate supplementation on anaerobic performance and body composition in American football players. Int J Sport Nutr.

[11] Ostojic S.M., Ahmetovic Z. (2009). The effect of 4 weeks treatment with a 2-gram daily dose of pyruvate on body composition in healthy trained men. Int J Vitam Nutr Res.

[12] Kalman D., Colker C.M., Stark R., Minsch A., Wilets I., Antonio J. (1998). Effect of pyruvate supplementation on body composition and mood. Curr Ther Res Clin Exp.

[13] Stanko R.T., Robertson R.J., Galbreath R.W., Reilly J.J., Greenawalt K.D., Goss F.L. (1990). Enhanced leg exercise endurance with a high-carbohydrate diet and dihydroxyacetone and pyruvate. J Appl Physiol (1985).

[14] Stanko R.T., Robertson R.J., Spina R.J., Reilly J.J., Greenawalt K.D., Goss F.L. (1990). Enhancement of arm exercise endurance capacity with dihydroxyacetone and pyruvate. J Appl Physiol (1985).

[15] Ebersole K.T., Stout J.R., Eckerson J.M., Housh T.J., Evetovich T.K., Smith D.B. (2000). The effect of pyruvate supplementation on critical power. J Strength Condit Res.

[16] Morrison M.A., Spriet L.L., Dyck D.J. (2000). Pyruvate ingestion for 7 days does not improve aerobic performance in well-trained individuals. J Appl Physiol (1985).

[17] Maughan R.J., Burke L.M., Dvorak J. (2018). IOC consensus statement: dietary supplements and the high-performance athlete. Int J Sport Nutr Exerc Metabol.

[18] Yang Y.P., Qiu J.Q., Wang M.Y. (2022). Effects of sodium pyruvate supplementation on repeated sprint exercise performance and recovery in male college soccer players: a randomized controlled trial. Ann Palliat Med.

[19] Waterhouse C., Keilson J. (1969). Cori cycle activity in man. J Clin Invest.

[20] Olek R.A., Luszczyk M., Kujach S. (2015). Single pyruvate intake induces blood alkalization and modification of resting metabolism in humans. Nutrition.

[21] LaNoue K., Nicklas W.J., Williamson J.R. (1970). Control of citric acid cycle activity in rat heart mitochondria. J Biol Chem.

[22] Constantin-Teodosiu D., Simpson E.J., Greenhaff P.L. (1999). The importance of pyruvate availability to PDC activation and anaplerosis in human skeletal muscle. Am J Physiol.

[23] Poole R.C., Halestrap A.P. (1993). Transport of lactate and other monocarboxylates across mammalian plasma membranes. Am J Physiol.

[24] Zhou F.Q. (2005). Pyruvate in the correction of intracellular acidosis: a metabolic basis as a novel superior buffer. Am J Nephrol.

[25] Mongan P.D., Fontana J.L., Chen R., Bunger R. (1999). Intravenous pyruvate prolongs survival during hemorrhagic shock in swine. Am J Physiol.

[26] Olek R.A., Kujach S., Wnuk D., Laskowski R. (2014). Single sodium pyruvate ingestion modifies blood acid-base status and post-exercise lactate concentration in humans. Nutrients.

[27] Mallet R.T., Olivencia-Yurvati A.H., Bunger R. (2018). Pyruvate enhancement of cardiac performance: cellular mechanisms and clinical application. Exp Biol Med (Maywood).

[28] Andres S., Ziegenhagen R., Trefflich I. (2017). Creatine and creatine forms intended for sports nutrition. Mol Nutr Food Res.

[29] Kreider R.B., Jäger R., Purpura M. (2022). Bioavailability, efficacy, safety, and regulatory status of creatine and related compounds: a critical review. Nutrients.

[30] Holleman A.F. (1904). Notice sur l'action de l'eau oxygénée sur les acides α-cétoniques et sur les dicétones 1. 2. Recueil des Travaux Chimiques des Pays-Bas et de la Belgique.

[31] Olek R.A., Antosiewicz J., Popinigis J., Gabbianelli R., Fedeli D., Falcioni G. (2005). Pyruvate but not lactate prevents NADH-induced myoglobin oxidation. Free Radic Biol Med.

[32] Ziolkowski W., Wierzba T.H., Kaczor J.J. (2008). Intravenous sodium pyruvate protects against cerulein-induced acute pancreatitis. Pancreas.

[33] Olek R.A., Ziolkowski W., Kaczor J.J., Wierzba T.H., Antosiewicz J. (2011). Higher hypochlorous acid scavenging activity of ethyl pyruvate compared to its sodium salt. Biosci Biotechnol Biochem.

[34] Zhang X.M., Deng H., Tong J.D. (2020). Pyruvate-enriched oral rehydration solution improves glucometabolic disorders in the kidneys of diabetic db/db mice. J Diabetes Res.

[35] Vasquez-Vivar J., Denicola A., Radi R., Augusto O. (1997). Peroxynitrite-mediated decarboxylation of pyruvate to both carbon dioxide and carbon dioxide radical anion. Chem Res Toxicol.

[36] Dobsak P., Courderot-Masuyer C., Zeller M. (1999). Antioxidative properties of pyruvate and protection of the ischemic rat heart during cardioplegia. J Cardiovasc Pharmacol.

[37] Fedeli D., Falcioni G., Olek R.A. (2007). Protective effect of ethyl pyruvate on msP rat leukocytes damaged by alcohol intake. J Appl Toxicol.

[38] Hassel B. (2001). Pyruvate carboxylation in neurons. J Neurosci Res.

[39] Park J.W., Chun Y.S., Kim M.S., Park Y.C., Kwak S.J., Park S.C. (1998). Metabolic modulation of cellular redox potential can improve cardiac recovery from ischemia-reperfusion injury. Int J Cardiol.

[40] Spriet L.L., Howlett R.A., Heigenhauser G.J. (2000). An enzymatic approach to lactate production in human skeletal muscle during exercise. Med Sci Sports Exerc.

[41] Schantz P.G. (1986). Plasticity of human skeletal muscle with special reference to effects of physical training on enzyme levels of the NADH shuttles and phenotypic expression of slow and fast myofibrillar proteins. Acta Physiol Scand Suppl.

[42] Radak Z., Suzuki K., Posa A., Petrovszky Z., Koltai E., Boldogh I. (2020). The systemic role of SIRT1 in exercise mediated adaptation. Redox Biol.

[43] Ji L.L., Yeo D. (2022). Maintenance of NAD+ homeostasis in skeletal muscle during aging and exercise. Cells.

[44] Zhou L., Pinho R., Gu Y., Radak Z. (2022). The role of SIRT3 in exercise and aging. Cells.

[45] Gambini J., Gomez-Cabrera M.C., Borras C. (2011). Free [NADH]/[NAD(+)] regulates sirtuin expression. Arch Biochem Biophys.

[46] Ide K., Schmalbruch I.K., Quistorff B., Horn A., Secher N.H. (2000). Lactate, glucose and O2 uptake in human brain during recovery from maximal exercise. J Physiol.

[47] Dalsgaard M.K., Ide K., Cai Y., Quistorff B., Secher N.H. (2002). The intent to exercise influences the cerebral O(2)/carbohydrate uptake ratio in humans. J Physiol.

[48] Zilberter M., Ivanov A., Ziyatdinova S. (2013). Dietary energy substrates reverse early neuronal hyperactivity in a mouse model of Alzheimer's disease. J Neurochem.

[49] Koivisto H., Leinonen H., Puurula M. (2016). Chronic pyruvate supplementation increases exploratory activity and brain energy reserves in young and middle-aged mice. Front Aging Neurosci.

[50] Isopi E., Granzotto A., Corona C. (2015). Pyruvate prevents the development of age-dependent cognitive deficits in a mouse model of Alzheimer's disease without reducing amyloid and tau pathology. Neurobiol Dis.

[51] Zilberter Y., Gubkina O., Ivanov A.I. (2015). A unique array of neuroprotective effects of pyruvate in neuropathology. Front Neurosci.

[52] He Y., An J., Yin J.J. (2021). Ethyl pyruvate-derived transdifferentiation of astrocytes to oligodendrogenesis in cuprizone-induced demyelinating model. Neurotherapeutics.

[53] Wang J., Zhang S., Ma H. (2017). Chronic intermittent hypobaric hypoxia pretreatment ameliorates ischemia-induced cognitive dysfunction through activation of ERK1/2-CREB-BDNF pathway in anesthetized mice. Neurochem Res.

[54] Kao K.K., Fink M.P. (2010). The biochemical basis for the anti-inflammatory and cytoprotective actions of ethyl pyruvate and related compounds. Biochem Pharmacol.

[55] Fukushima M., Lee S.M., Moro N., Hovda D.A., Sutton R.L. (2009). Metabolic and histologic effects of sodium pyruvate treatment in the rat after cortical contusion injury. J Neurotrauma.

[56] Suh S.W., Aoyama K., Matsumori Y., Liu J., Swanson R.A. (2005). Pyruvate administered after severe hypoglycemia reduces neuronal death and cognitive impairment. Diabetes.

[57] Ullah N., Naseer M.I., Ullah I., Kim T.H., Lee H.Y., Kim M.O. (2013). Neuroprotective profile of pyruvate against ethanol-induced neurodegeneration in developing mice brain. Neurol Sci.

[58] Miao Y., Qiu Y., Lin Y., Miao Z., Zhang J., Lu X. (2011). Protection by pyruvate against glutamate neurotoxicity is mediated by astrocytes through a glutathione-dependent mechanism. Mol Biol Rep.

[59] Nakamichi N., Kambe Y., Oikawa H. (2005). Protection by exogenous pyruvate through a mechanism related to monocarboxylate transporters against cell death induced by hydrogen peroxide in cultured rat cortical neurons. J Neurochem.

[60] Ryou M.G., Choudhury G.R., Winters A., Xie L., Mallet R.T., Yang S.H. (2013). Pyruvate minimizes rtPA toxicity from in vitro oxygen-glucose deprivation and reoxygenation. Brain Res.

[61] Sheline C.T., Behrens M.M., Choi D.W. (2000). Zinc-induced cortical neuronal death: contribution of energy failure attributable to loss of NAD(+) and inhibition of glycolysis. J Neurosci.

[62] Zlotnik A., Gurevich B., Cherniavsky E. (2008). The contribution of the blood glutamate scavenging activity of pyruvate to its neuroprotective properties in a rat model of closed head injury. Neurochem Res.

[63] Zlotnik A., Sinelnikov I., Gruenbaum B.F. (2012). Effect of glutamate and blood glutamate scavengers oxaloacetate and pyruvate on neurological outcome and pathohistology of the hippocampus after traumatic brain injury in rats. Anesthesiology.

[64] Carvalho A.S., Torres L.B., Persike D.S. (2011). Neuroprotective effect of pyruvate and oxaloacetate during pilocarpine induced status epilepticus in rats. Neurochem Int.

[65] Kim J.B., Yu Y.M., Kim S.W., Lee J.K. (2005). Anti-inflammatory mechanism is involved in ethyl pyruvate-mediated efficacious neuroprotection in the postischemic brain. Brain Res.

[66] Yi J.S., Kim T.Y., Kyu Kim D., Koh J.Y. (2007). Systemic pyruvate administration markedly reduces infarcts and motor deficits in rat models of transient and permanent focal cerebral ischemia. Neurobiol Dis.

[67] He Y., An J., Yin J.J. (2019). Ethyl pyruvate enhances spontaneous remyelination by targeting microglia phagocytosis. Int Immunopharm.

